# Improving the qualitative indicators of apple juice by *Chitosan* and ultrasound

**DOI:** 10.1002/fsn3.925

**Published:** 2019-02-27

**Authors:** Saba Belgheisi, Reza EsmaeilZadeh Kenari

**Affiliations:** ^1^ Department of Food Science and Technology, Faculty of Food Industry and Agriculture Standard Research Institute (SRI) Karaj Iran; ^2^ Department of Food Science and Technology Sari Agricultural Sciences and Natural Resources University Sari Iran

**Keywords:** clarifying agents, fruit juice, nonthermal processing, quality

## Abstract

Today's consumers desire for tasty, nutritious, and safe food products, so researchers are looking for new ways in which little heat or no heat at all is used for processing. This study was to evaluate the effect of treatment using an ultrasonic bath (for 15, 30, and 60 min at 40 and 60°C) and ultrasonic probe (for 10, 15, and 20 min at 40 and 60°C), treatment with *Chitosan,* and combination of them on the quality of apple juice that includes physicochemical features (pH, acidity, total solid matter), total polyphenol, total antioxidant capacity, the cloud point, and color values of Hunter (L*, a*, b*) in the treated samples and comparing them with each other. The results showed that ultrasound has no effect on the pH and acidity, while the total solid of ultrasound treatment was higher than controls in combination with *Chitosan* (*p* < 0.05). Total polyphenols of apple juice samples treated by ultrasonic probe are higher than an ultrasonic bath (*p* < 0.05). The total antioxidant capacity has improved in treatments (*p* < 0.05). According to the results there is a significant difference between the cloud point of control samples and *Chitosan* (*p* < 0.05). The L* (brightness) increased in ultrasonic probe method and had a significant decline in *Chitosan*treatment (*p* < 0.05). Findings from this study suggest that the use of ultrasound treatment in the production of apple juice can improve quality factors, and in this regard, ultrasonic probe is more effective.

## 1 INTRODUCTION

Apples are a rich source of bioactive compounds such as flavonoids, phenolic acids, carotenoids, fiber, minerals, and vitamins. These compounds play an important role in protecting the body from many diseases such as cardiovascular disease, impaired immune system, asthma, and diabetes (Boyer and Liu, 2004; Castro Domingues, Faria Junior, Silva, Cardoso, & Miranda Reis, 2012). Apple juice is produced in two forms of clear and cloud in the industry. Transparency in the fruit juice industry takes place more by thermal, enzymatic, centrifuges, and using absorbent and coagulation factors such as bentonite, silica, and *Chitosan*. *Chitosan* (*Chitin Deacetylated*) is the combination of nontoxic and biodegradable, natural, and eco‐friendly that is mainly from shrimp shells and due to the poly cationic natures considered as an active coagulator factor that can bring out the pectic substances that cause water turbidity of apples. Although thermal methods increase the food durability but in some cases, it causes nutritional value loss. The application of ultrasound in the food industry is a useful and attractive tool due to high efficiency, short time, easy, cost and energy saving, a method of "eco‐friendly" as defined in Ashokkumar et al. (2008) and Castro Domingues et al. (2012). Ultrasound equipment in laboratories is either baths or ultrasonic probe. Ultrasonic bath is economically affordable, and handling equipment can be easily carried out. The other type of ultrasound equipment is ultrasound probe that has a higher intensity and power because the material is applied at the micro level, and electrical failure occurs less frequently because probe is placed directly into the reaction vessel. The aim of this study was to evaluate the effect of treatment by ultrasound (bath and probes), treatment with *Chitosan* alone, and the combination of ultrasound treatments (bath and probe) and *Chitosan* on the quality of apple juice containing features like physicochemical (pH, acidity, total solids), total polyphenol, total antioxidant capacity, the cloud point, and Hunter color values (L*, a*, b*) in the treated samples and to compare them with each other.

## 2 MATERIAL AND METHODS

### 2.1 Ingredients

Yellow apple variety of Golden Delicious was purchased from the market. Reagents and chemicals were prepared from Merck (Darmstadt, Germany) and consumption solvent was prepared from Barcelona, Spain, with the highest purity.

### 2.2 Chemicals

Commercial *Chitosan* (average molecular weight), buffer 4, buffer 7, hydrochloric acid, soda, methanol, gallic acid, Folin–Ciocalteau reagent (Folin–Ciocalteu), sodium, sulfuric acid, sodium phosphate, ammonium molybdate, and ascorbic acid.

### 2.3 Preparation of apple juice

Fresh apples were purchased from the local market. Apples were washed with water, dried with paper towels, and then were cut into four pieces with a stainless steel knife, while seeds and stems were separated from the apples. Apple juice was extracted using a domestic juice and was smoothed using a cleaning cloth. Control samples were tested in order to the pasteurization at 71°C for 6 s and stored in the refrigerator (Abid et al., 2013).

### 2.4 Treatment with *Chitosan*

Fifty milliliter of apple juice was poured in 250 ml Erlenmeyer flasks, and 5 ml commercial *Chitosan* 7.0% was added in water (*Chitosan* with average molecular weight). The Erlenmeyer flasks were incubated for 2 hr at 40°C. The samples were left at room temperature for 12 hr, finally were smoothen with Whatman filter paper (Chemat, Huma, & Khan, 2011), were heated in order to pasteurize for 6 s at 71°C, and were stored in the refrigerator until the testing (Erkhan‐Koc, Turkyilmaz, Yemis, & Ozkan, 2013; Gómez, Welti‐Chanes, & Alzamora, 2011; Maghsoudlou, Zabihi, & Alami, 2014; Tastan & Baysal, 2015).

### 2.5 Ultrasound treatment

Ultrasound was carried out in accordance with Table 1 (Abid et al., 2014,2013).

**Table 1:** Ultrasound treatment conditions

Conditions	Samples	Temperature(°C)	Time (min)	Power (watt) (%)	Frequency (kHz)	Power density (W/cm^3^)
Control	Fresh	–	–	–	–	–
Ultrasound (Bath)	USB40‐15	40	15	100	37	0.02
	USB40‐30	40	30	100	37	0.02
	USB40‐60	40	60	100	37	0.02
	USB60‐15	60	15	100	37	0.02
	USB60‐30	60	30	100	37	0.02
	USB60‐60	60	60	100	37	0.02
Ultrasound (Probe)	USP40‐10	40	10	25	20	0.058
	USP40‐15	40	15	25	20	0.058
	USP40‐20	40	20	25	20	0.058
	USP60‐10	60	10	40	20	0.088
	USP60‐15	60	15	40	20	0.088
	USP60‐20	60	20	40	20	0.088
*Chitosan*	Fresh	–	–	–	–	–
*Chitosan + *ultrasound (bath)	Chi+USB40‐15	40	15	100	37	0.02
	Chi+USB40‐30	40	30	100	37	0.02
	Chi+USB40‐60	40	60	100	37	0.02
	Chi+USB60‐15	60	15	100	37	0.02
	Chi+USB60‐30	60	30	100	37	0.02
	Chi+USB60‐60	60	60	100	37	0.02
*Chitosan + *ultrasound (probe)	Chi+USP40‐10	40	10	25	20	0.058
	Chi+USP40‐15	40	15	25	20	0.058
	Chi+USP40‐20	40	20	25	20	0.058
	Chi+USP60‐10	60	10	40	20	0.088
	Chi+USP60‐15	60	15	40	20	0.088
	Chi+USP60‐20	60	20	40	20	0.088

### 2.6 Total solids (Brix)

The total solid content was measured at 20 ± 0.5°C using a refract meter (Abid et al., 2013).

### 2.7 pH

pH was measured at 20 ± 0.5°C. pH meter was calibrated with a solution of a commercial buffer 4 and 7 (Abid et al., 2013).

### 2.8 Titratable acidity

Ten milliliter of the sample was poured into 250 ml of the beaker, and 90 ml of distilled water was added. The solution was titrated with a profit of 1/N to the endpoint of pH = 2.8 ± 0.1. Titratable acidity was obtained from the following equation (AOAC, 1999):

$$\text{Acidity}=\frac{\text{titratesize}\times 100\times \text{acidfactor}\times \text{profitnormality}}{\text{samplesizeinml}}$$

### 2.9 Total polyphenol content

The total content of phenolic was carried out using two methods of spectrophotometry and Folin–Ciocalteau. Gallic acid was used as a standard (Boyer & Liu, 2004).

### 2.10 Total antioxidant capacity

Apple juice total antioxidant capacity was measured in accordance with a procedure done by Oboh and Ademosun (2012). Ascorbic acid was used as standard, and the antioxidant capacity was measured compared to it.

### 2.11 The cloud point

The procedure was done with a little modification in accordance with the Versteeg, Rombouts, Spaansen, and Pilnik (2006).

### 2.12 Color

Sample color was analyzed using a colorimeter at Hunter Laboratory. The device was calibrated with white reference. Color values were read in terms of CIE L* a* b* system that L* is (white or light to dark ratio), a* (red–green ratio), and b* (yellow–blue ratio) (Abid et al., 2013).

### 2.13 Statistical analysis

Statistical analysis of treatments was carried out by the analysis of variance (ANOVA) using SPSS_16_ software. A significant difference between means was determined at 0.05 by Duncan test.

## 3 RESULTS AND DISCUSSION

### 3.1 Determination of pH, acidity, and total solids changes

**Figure 1:**
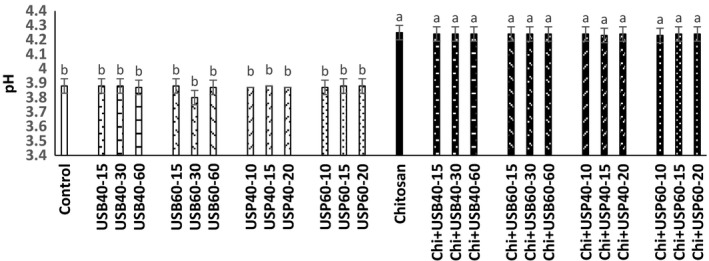
The effect of ultrasound and *Chitosan*on pH of apple juice. Values with different letters are significantly different in each column (*p* < 0.05)

**Figure 2:**
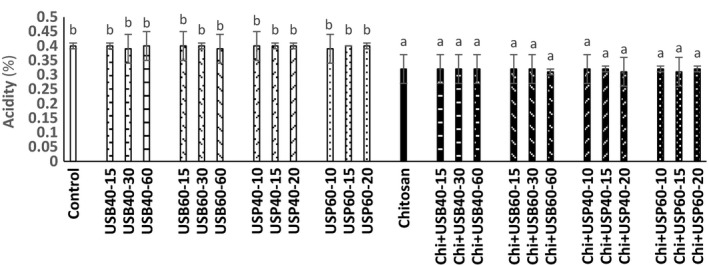
The effect of ultrasound and *Chitosan*on acidity apple juice. Values with different letters are significantly different in each column (*p* < 0.05)

**Figure 3:**
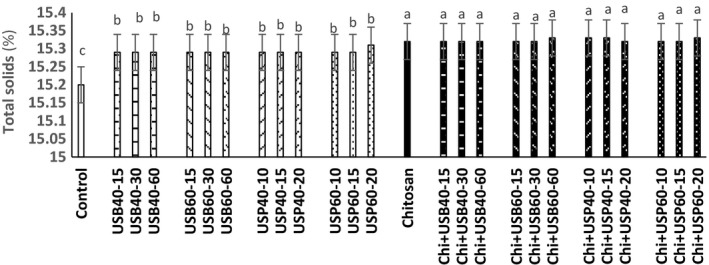
The effect of ultrasound and *Chitosan*on total solid content of apple juice. Values with different letters are significantly different in each column (*p* < 0.05)

The effect of ultrasound and *Chitosan* on pH, acidity, and total solids (Brix) of apple juice samples is shown in Figures 1, 2, 3. There was no difference between the pH and acidity of ultrasound treatments (bath and probe) and controls (*p* > 0.05). The pH and acidity of the samples treated with *Chitosan* had no significant difference with combination of ultrasound + *Chitosan* treatment (*p* > 0.05).

These results are consistent with the findings of Walkling‐Ribeiro, Noci, Cronin, Lyng, and Morgan (2009) that observed no significant effect of combined treatment of electric pulses and ultrasound on pH, acidity, and total solid contents of orange juice. The findings of Abid et al. (2013) also showed that ultrasound does not cause significant changes in pH, acidity, and total solid content in apple juice. Results obtained by Tiwari, Patras, Brunton, Cullen, and O'Donnell (2010) did not show significant differences in the pH, acidity, and total solids during grape juice ultrasound treatment. pH of *Chitosan* samples was higher than control and ultrasound (*p* < 0.05). This effect can be attributed to the nature of poly cationic *Chitosan* that has a linking property with acid and excluded acidic compounds from the environment. Data from the survey of *Chitosan* and ultrasound on the total solids of apple juices showed that samples treated with ultrasound are higher in total solids than the control (*p* < 0.05). The total amount of solid material samples treated with *Chitosan* and ultrasound +* Chitosan* treatments have higher total solids than other treatments (control and ultrasound), respectively (*p* < 0.05). However, according to Abid et al. (2013) ultrasound treatments do not have significant effect on the total solids of apple juice. Increase in the total solids compared to the ultrasound + *Chitosan*treatments could be due to the effect of ultrasonic cavitation that turns macromolecules into smaller units. According to Aadil et al. (2014), there was no significant difference in the pH, acidity, and total solids of grapefruit juice treated with ultrasound in comparison with the control sample (*p* > 0.05).

### 3.2 Assessment of total polyphenol and total antioxidant capacity changes

**Figure 4:**
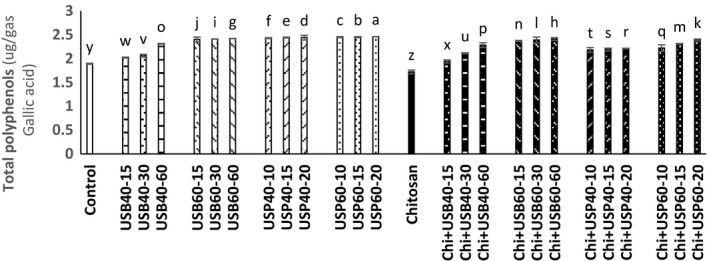
The effect of ultrasound and *Chitosan* on total polyphenols of apple juice. Values with different letters are significantly different in each column (*p* < 0.05)

**Figure 5:**
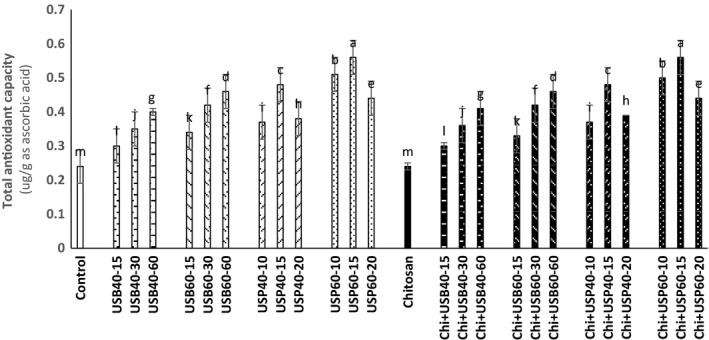
The effect of ultrasound and *Chitosan* on total antioxidant capacity of apple juice. Values with different letters are significantly different in each column (*p* < 0.05)

The results of the ultrasound and the effect of *Chitosan* on total polyphenols and total antioxidant capacity of apple juice samples are shown in Figures 4 and 5. According to data obtained, there was no significant difference between ultrasound treatments and control samples (*p* < 0.05). Total polyphenols of ultrasound treatment were higher than at 40°C. Total polyphenols of apple juice samples treated by ultrasonic probe were higher than an ultrasonic bath (*p* < 0.05).

Abid et al. (2014) studied the effects of ultrasound on the total polyphenol content in apple juice and observed significant difference between ultrasound and control samples (*p* < 0.05), and ultrasound treatments had higher total polyphenols. Also, increasing temperature from 40 to 60°C had a significant effect on these compounds and ultrasound probe is more effective than an ultrasonic bath at 60°C. The findings of Abid et al. (2013) showed a significant increase in total phenols of fruit juices treated with ultrasound for 30, 60, and 90 min compared with the control. The probable reason for the increasing significance of such phenolic compounds is probably due to escalating certain inappropriate cell wall conditions caused by cavitations and rapidly changing fluid pressure due to shear forces applied during ultrasound that may release some polyphenol compounds and ultimately increase its availability in the juice. Hydroxyl radical's connection to aromatic ring of phenolic compounds during ultrasound through bubble blast may increase them in the apple juice. It was also reported that the increase in the antioxidant capacity of phenolic compounds is attributed to addition of the second group of hydroxyl to *ortho* and *para* positions. Increasing polyphenol oxidase activity in the ultrasound treatment may be another reason for the increase of these compounds. All these results suggest that ultrasound of apple juice is beneficial for consumers from a commercial standpoint and from the standpoint of nutrition.

According to the results presented in Figure 5, there is a significant difference between the treatments (*p* < 0.05). The total antioxidant capacity of ultrasound and ultrasound + *Chitosan* treatments was higher than control.

This finding is similar to the findings obtained by Abid et al. (2013) in which a significant increase in total antioxidant capacity of apple juice was observed by ultrasound treatment. Total polyphenols of all treatments are less than treatments without *Chitosan*(*p* < 0.05). These findings are similar to results obtained by Erkan‐Koc et al. (2013) that in clarification of pomegranate juice with clarification elements based on the polysaccharide conclude that loss of phenolic compounds during clarification is the result of oxidation of phenolic and clarifying agents based on polysaccharides such as *Chitosan* lowering effect on the amount of phenolic as precipitator. Oszmianski and Wojdyło (2007) reported that *Chitosan* has no effect on antioxidant activity of apple juice, and our results are similar to their findings. So that the ultrasound treatment increased the total antioxidant capacity of apple juice samples; however, *Chitosan* had no effect on the total antioxidant activity of apple juice (*p* < 0.05). An increase in temperature from 40 to 60°C and the ultrasonic probe were more effective in increasing total antioxidant capacity (*p* < 0.05). Increase in total antioxidant capacity can be because of increase in ascorbic acid and phenolic compounds resulting from cavitations over the ultrasound of apple juice that increases extracting such compounds. Polyphenolic compounds have a high antioxidant capacity.

### 3.3 Determination of cloud point and color changes

**Figure 6:**
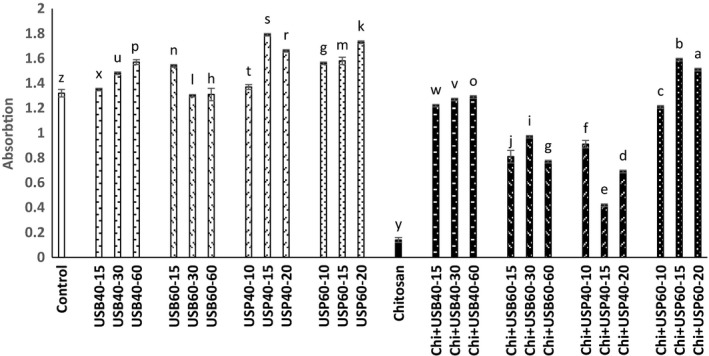
Effect of ultrasound and *Chitosan* on the cloud point. Values with different letters are significantly different in each column (*p* < 0.05)

**Figure 7:**
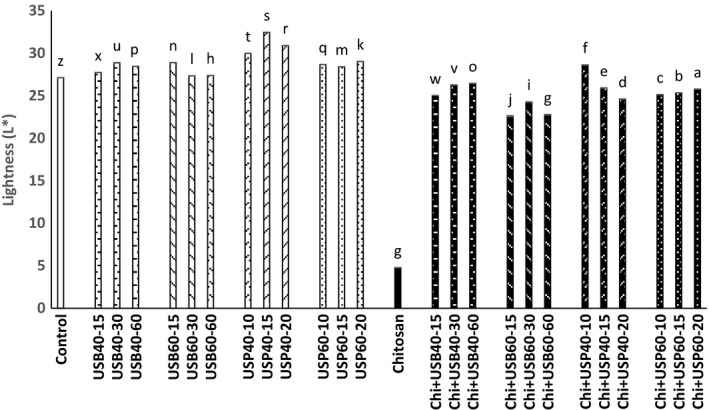
Effect of ultrasound and *Chitosan* on L*. Values with different letters are significantly different in each column (*p* < 0.05)

**Figure 8:**
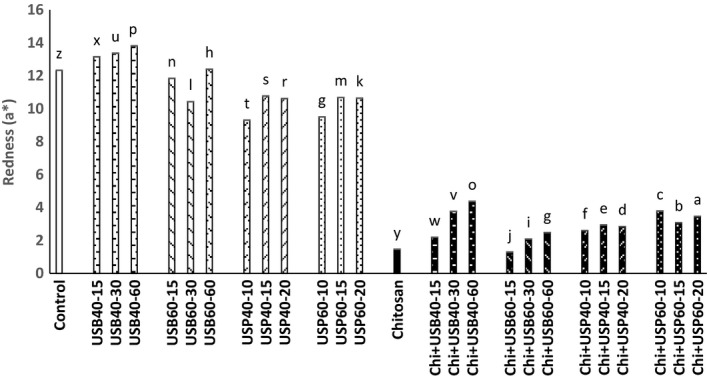
Effect of ultrasound and *Chitosan* on a*. Values with different letters are significantly different in each column (*p* < 0.05)

**Figure 9:**
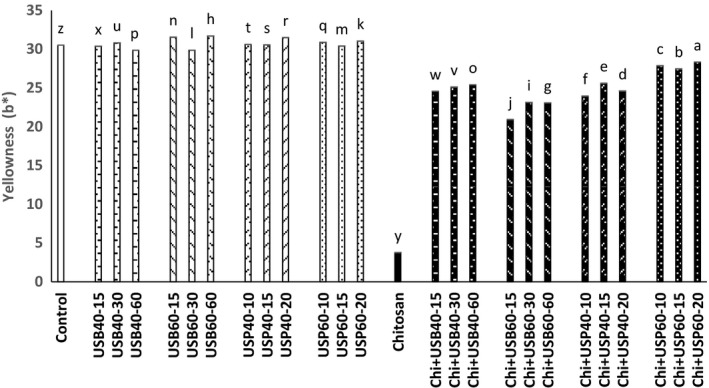
Effect of ultrasound and *Chitosan* on b*. Values with different letters are significantly different in each column (*p* < 0.05)

The results obtained about the effect of ultrasound and *Chitosan*on the cloud point and color (L*, a*, b*) is shown in Figures 6, 7, 8, 9.

According to the results, there is a significant difference between the cloud point of control samples and *Chitosan*(*p* < 0.05). The cloud point in ultrasonic probe has increased compared to an ultrasonic bath (*p* < 0.05). The least amount of cloud point was for *Chitosan* treatment (0.14 ± 0.02), and the most cloud point was for the samples treated with ultrasonic probe for 15 min at 40°C.

In accordance with the findings of Abid et al. (2013), ultrasound treatment significantly increased the cloud point in apple juice (*p* < 0.05). Increase in cloud point of ultrasound treatments compared with control samples and *Chitosan* + ultrasound treatments is probably due to the high‐pressure gradient caused by cavitation during ultrasound treatment. That deplete colloids, dispersion and decomposition of macromolecules into smaller units, and juices are well homogeneous and more stable. Some studies suggest that ultrasound reduced molecular weight of pectin by breaking the linear molecule and therefore form a weak network. Increases in temperature and time do not have a significant effect on the cloud point (*p* < 0.05). According to research carried out by Fatih Ertugay and Baslar (2014) on a microscopic scale, ultrasound increases cloud level and stabilize fruit juice by crushing coarse particles in the juice and creates a stable suspension so it will improve physical appearance of apple juice. Based on research of Abid et al. (2014), the effect of ultrasound treatments and electric pulses was significant on the cloud point of grapefruit. The amount of cloud in ultrasound and combination treatments significantly increased compared with control samples. This could be due to breaking down larger molecules into smaller due to the applied gradient pressure because of cavitations and increase in level that improve cloud points in fruit juices. Tiwari, Muthukumarappan, O'Donnell, and Cullen (2008) also observed that the ultrasound exacerbates the cloud point of orange juice. The results of the L*, a*, b* indicate that there is a significant difference between the control group and *Chitosan* (*p* < 0.05). The amount L* (brightness) increases in the ultrasound probe compared to an ultrasound bath and had a significant reduction in *Chitosan* treatment compared to control and other ultrasound treatment (*p* < 0.05).

Unwanted particle deposition in apple juice due to the ultrasound treatment is likely responsible for the increase in L*. Tiwari et al. (2008) reported that an increase in the amount of L* is probably due to an increase in the amount of cloud point of juice under ultrasounds that leading to better homogenization. Change in the color of apple juice treated with ultrasound alone or in combination with *Chitosan* is possibly due to the effects of time and temperature variables. The amount of a* (red) in *Chitosan* + ultrasound treatments had a significant reduction compared to ultrasound treatments (*p* < 0.05) and was higher at 40°C than 60°C. The amount of b* (yellow) of *Chitosan* + ultrasound treatments had a significant reduction compared to ultrasound treatments (*p* < 0.05) and was higher at 40°C than 60°C. b* samples treated with *Chitosan* were lower than all other treatments (*p* < 0.05).

Studies of Abid et al. (2014) showed a significant increase in L* and b* and a drop in a* of treated apple juice using ultrasound method (bath and probe). Abid et al. (2013) concluded that ultrasound treatment resulted in significant changes in the color of the treated samples compared to the control. Also, according to Tiwari et al. (2010), during the heating grape juice, compared to the control treatment at all times and ultrasound levels, an increases in brightness (L*) were observed. Aadil et al. (2014) investigated the effects of ultrasound on grapefruit juice, and results show that that brightness, redness, and yellowness remain unchanged after the ultrasound and pulse electric treatment and the combination of them compared to control. Changes in color during the ultrasound cavitations cause physical, chemical, and biologic changes such as increase in the speed of diffusion and decomposition of susceptible particles like enzymes and microorganisms. Tastan and Baysal (2015) concluded that the increase in the concentration of *Chitosan* may increase a* as a clarification factor in the production of pomegranate juice. The interaction of *Chitosan*concentration and process time has a positive effect on a*.

## 4 CONCLUSION

Findings from this study suggest that the use of ultrasound treatment in the production of apple juice can improve the quality factors, and in this field, ultrasound probe is more effective than ultrasound bath. The ultrasound treatment significantly improves the phenolic compounds, total antioxidant capacity without affecting the physicochemical parameters of pH and acidity of apple juice. The cloud point and chroma Hunter in samples treated with ultrasound have improved compared to *Chitosan*. Therefore, it is suggested to use new technology of ultrasound to improve the quality of apple juice from the standpoint of the consumer health. However, further research is needed to determine the effect of ultrasound on the sensory and functional properties of apple juice.

## CONFLICT OF INTEREST

None declared.

## ETHICAL STATEMENT

This work does not involve any human or animal studies.
